# Should We Be Worried about Smartphone Addiction? An Examination of Canadian Adolescents’ Feelings of Social Disconnection in the Time of COVID-19

**DOI:** 10.3390/ijerph19159365

**Published:** 2022-07-30

**Authors:** Natasha Parent, Bowen Xiao, Claire Hein-Salvi, Jennifer Shapka

**Affiliations:** Educational and Counselling Psychology and Special Education, The University of British Columbia, Vancouver, BC V6T 1Z4, Canada; bowenxiao3@cmail.carleton.ca (B.X.); clairevaleria@hotmail.com (C.H.-S.); jennifer.shapka@ubc.ca (J.S.)

**Keywords:** smartphone addiction, COVID-19, mental health, adolescents, social disconnection

## Abstract

As the COVID-19 global pandemic limited face-to-face social contact, mental health concerns increased for adolescents. Additionally, many adolescents turned to technology to communicate with their peers, which also raised concerns about adolescent smartphone addiction. However, research has yet to examine how mental health and technology engagement are related to adolescents’ feelings of social connection—an important developmental predictor of wellbeing across the lifespan. Specifically, little is known regarding the relative risk of adolescents’ mental health concerns, a known risk factor for social disconnection and isolation and smartphone addiction in contributing to feelings of social disconnection in the time of COVID-19. The present study investigated how mental health outcomes and smartphone addiction contributed to Canadian adolescents’ (*n* = 1753) feelings of social disconnection during COVID-19. Between October 2020 and May 2021, data were collected from five secondary schools in and around the lower mainland of British Columbia using an online-administered self-report questionnaire. Adolescents responded to questions about their smartphone addiction, internalizing problems, and an open-ended question about their feelings of connection to others. Findings from logistic regression analyses indicated that depression was a predictor of feeling socially disconnected: however, smartphone addiction was not associated with feelings of social disconnection during COVID-19. Implications of these findings can help inform the development of prevention programs targeting adolescents at risk for social disconnection in times of increased social isolation (e.g., a global pandemic). Specifically, these findings suggest that adolescents higher in depressive symptoms, and not those higher in smartphone addiction, are the ones most at risk.

## 1. Introduction

The global COVID-19 pandemic has led to drastic changes in the lives of Canadian youth. Indeed, government efforts to control the spread of COVID-19, such as limiting physical social interactions, have included periodic school closures, the cancelation of extracurricular activities, and an overall decrease in in-person opportunities to socialize. While this context has been challenging for most Canadians, the effects of this decreased socialization may be particularly detrimental for adolescents, for whom the need for social connection is developmentally rooted and exacerbated [1]. As adolescents faced limited opportunities to connect with others in-person in the time of COVID-19, many turned to technology to establish and maintain their peer relationships [2,3]. Unfortunately, this increased reliance on technology during the pandemic has contributed to heightened concerns around screen use and the negative impacts of smartphone addiction (this article uses the term “smartphone addiction” to refer to the compulsive and excessive use of smartphones, which results in negative consequences, also known as problematic smartphone use; while the authors typically recommend avoiding using the term “smartphone addiction” to avoid stigmatizing what are often adaptative and normative behaviors towards smartphones, this language is used in the current study to parallel the growing moral panic around smartphone use) [4,5], which has been linked to increased stress, anxiety, depression, and loneliness [6]. While an emerging body of work has examined adolescents’ wellbeing [2,7,8,9] and smartphone addiction [10] during the pandemic, the social implications of the pandemic relative to increased screen use and mental health concerns have yet to be explored. Thus, the current study aimed to address this gap by examining the role of adolescents’ mental health and smartphone addiction in contributing to feelings of social disconnection during COVID-19.

Regarding COVID-related mental health concerns among children and adolescents, findings from the Canadian Perspectives Survey indicated that only 42% of youth aged 15–24 reported having excellent or very good mental health during the initial phases of COVID-19, compared to 62% in 2018 [9]. Additionally, a study conducted in Ontario found that the population-level demand for mental health care for those aged 3–17 increased above expected levels through the fall of 2020 and continued to rise until early 2021 [8]. More importantly, these increased rates of mental health concerns may pose important risks for adolescents’ healthy development. Specifically, as adolescents experience increasing levels of depression, anxiety, or stress, they may withdraw from their peers and become more socially isolated [11], which places them at increased risk for a multitude of negative outcomes, including loneliness, aggression, criminality, substance use, school dropout, and even more internalizing problems, e.g., depression and anxiety [12,13,14,15].

Alongside the increasing concerns around adolescents’ mental health, the pandemic also took the existing moral panic around children and screens to new heights. Parents, educators, and researchers alike were worried that the pandemic would contribute to increased rates of smartphone addiction among teens and that this, in turn, would lead to maladaptive outcomes, including feelings of social disconnection and loneliness [4,5]. Importantly, smartphone addiction is not the same as screen time or overall smartphone use. Rather, smartphone addiction refers to a compulsive pattern of smartphone use that disrupts daily life [16], and has been linked with loneliness, depression, anxiety, and stress [17]. In this way, smartphone addiction is a problematic pattern of behaviors aimed towards the smartphone that results in negative consequences. In contrast to this, adolescents’ smartphone use, more broadly, has been found to be an adaptive means of fostering feelings of connection with others during the pandemic. For example, a study conducted in Ontario found that connecting with others virtually was an effective way for adolescents to combat feelings of loneliness during the initial COVID-19 lockdown [2]. These findings are supported by the work of Anderson and Jiang [18], who found that the use of technology to establish relationships and connect with others was a strong motivation for technology engagement among adolescents, as well as findings by Michikyan and Suárez-Orozco [19], showing that youth often turn to social media as a means of fostering feelings of social connection and intimacy. While these studies point to a well-intentioned use of smartphones, findings from Li et al. [10] showed that during COVID-19, even this type of adaptive smartphone use resulted in increased rates of smartphone addiction. Specifically, Li et al. [10] found that Chinese adolescents who reported feeling lonelier were more likely to report higher levels of smartphone addiction, which they attributed to adolescents’ increased use of smartphones to escape the loneliness of the pandemic reality. In other words, it appears that what began as an adaptive use of smartphones to connect with others during COVID-19, often developed into smartphone addiction among adolescents who were experiencing loneliness. Given the established link between smartphone addiction and loneliness [10,17], it is possible then that, during COVID-19, lonely individuals may have sought social connection through their devices, which in turn may have placed them at increased risk for smartphone addiction, thus, further exacerbating their feelings of isolation and social disconnection.

The current study—an empirical examination of how mental health and smartphone addiction were related to social disconnection during the pandemic—could help us understand the relative risk of smartphone addiction in contributing to adolescents’ wellbeing during COVID-19. Given the ongoing nature of the pandemic, and the concomitant concerns about the state of adolescents’ wellbeing, understanding the impact of technology engagement, and smartphone addiction, in particular, is an important gap to address. Rooted in the understanding that humans have a fundamental and intrinsic need for social connection [20], this work investigated the relative risk of empirically established risk factors (i.e., mental health and smartphone addiction) in predicting adolescents’ social disconnection. Based on past research [11,21,22,23], we expected that depression, anxiety, and stress would be positively associated with social disconnection during the pandemic. However, given the potential for high levels of smartphone use to function as an adaptive means of connecting with others in the time of COVID-19 [2,3], alongside the established relationship between smartphone addiction and loneliness [10,17], we were interested to explore how smartphone addiction was related to social disconnection during COVID-19.

## 2. Methods

### 2.1. Procedures

The current study obtained ethical approval from the institutional review board of The University of British Columbia. Participants were recruited from five secondary schools across two public school districts in British Columbia, Canada, through a combination of convenience and snowball sampling. All students from the participating schools were invited to take part in the study provided that their parent(s) or guardian(s) did not oppose their participation (i.e., passive consent). Prior to their participation, students also provided active informed assent. As a recruitment incentive, all participating school’s students were entered into a draw for a chance to win a smartphone. Data were collected separately at each school between the months of October 2020 and May 2021. All self-report questionnaires were completed online through Qualtrics (www.qualtrics.com) during school hours. Participants were informed that if the study upset them or if they did not want to participate for any reason, they could withdraw at any time.

### 2.2. Methodological Framework

This study employed a concurrent mixed-methods design [24]. That is, both the quantitative and qualitative data were simultaneously collected, combined, and analyzed. Using this approach, we identified themes derived from the qualitative analysis and, subsequently, integrated them into the quantitative analysis, allowing for a more thorough understanding of adolescents’ feelings of social connection in the time of the pandemic, as well as the examination of adolescents’ smartphone addiction and mental health in predicting social disconnection. Accordingly, the data for this study consisted of responses to self-report questionnaires and an open-ended survey question.

### 2.3. Participants

Participants were *N* = 2666 (1393 boys; *M*_age_ = 15.13 years; *SD =* 1.47 years) high school students between the ages of 11 and 20 (grades 8–12). The sample was ethnically diverse, with approximately 28.9% self-identifying as East Asian (see Table 1).

### 2.4. Measures

#### 2.4.1. Demographics

Participants were asked to self-report their school, age, grade, gender, ethnicity, language(s) spoken at home, and whether or not they were born in Canada.

#### 2.4.2. Smartphone Addiction

Smartphone addiction was measured using the Smartphone Addiction Scale-Short Version (SAS-SV) [25]. This measure comprised of 10 items that were rated on a 6-point Likert scale, ranging from 1 (strongly disagree) to 6 (strongly agree). Sample items included “I use my phone longer than intended” and “I feel impatient and irritated when I am not holding my phone.” A composite variable was created by calculating the average score across all items. This measure has been shown to have good psychometric properties [26]. The validity of the SAS-SV scale has also been consistently demonstrated. Specifically, it correlates strongly with the smartphone addiction proneness scale (SAPS) and the internet addiction test (IAT) [25,27,28]. It has also been linked with current smoking, weekly to daily alcohol drinking, and physical inactivity among young adults [29].

#### 2.4.3. Internalizing Problems

To assess their mental health, adolescents completed the Depression, Anxiety, and Stress Scale (DASS) [21,30]. The DASS-21 consists of 3 subscales: Depression (7 items, e.g., “I couldn’t seem to experience any positive feeling at all”), Anxiety (7 items, e.g., “I was aware of dryness of my mouth”), and Stress (7 items, e.g., “I found it hard to wind down”). Each item was rated on a 4-point Likert scale, ranging from 0 (did not apply to me at all) to 3 (applied to me very much or most of the time). Sum scores were computed by adding up the scores on the items per (sub)scale and multiplying them by 2. These cut-off scores were derived from a set of severity ratings, proposed by Lovibond and Lovibond [30]. Specifically, scores 0–14 were labeled as 1 (normal), scores 15–18 were labeled as 2 (mild), scores 19–25 were labeled as 3 (moderate), scores 26–33 were labeled as 4 (severe), and scores > 34 were labeled as 5 (extremely severe). This measure has been shown to have good psychometric properties [31].

#### 2.4.4. Feelings of Social Connection

In order to assess how the COVID-19 global pandemic impacted participants’ ability to connect with others, participants were asked an open-ended question designed specifically for the current study. Participants were asked to describe in their own words “how you feel personally and how you get along with and connect with others since the start of COVID-19”.

### 2.5. Data Analysis

Participant responses to the open-ended question were analyzed using thematic content analysis in Microsoft Excel (2022). Following this, quantitative analyses (i.e., preliminary analyses and binary logistic regression) were conducted using SPSS 22.0. For the binary logistic regression, gender, age, school, and ethnicity were entered as covariates in the first step to control for individual differences. In the second step, depression, anxiety, and stress were entered as a block. In the third step, smartphone addiction was added as a block. For each variable, this procedure produced odd ratios with 95 percent confidence intervals. Following this, factor blocks were entered sequentially, and differences in the −2 log likelihood values were checked against chi-square tables at each step to see if adding the new block of variables improved the model fit.

## 3. Results

The initial sample comprised of 2666 high school students. For the purpose of this study, only those who provided a response to the open-ended question about their feelings of social connection during the COVID-19 pandemic (response rate = 65.8%) were included in the final sample (N = 1753, 859 boys; *M*_age_ = 15.11 years; *SD* = 1.46 years). Participant responses to the open-ended questions were analyzed using thematic content analysis, leading to the identification of three broad thematic categories: (1) feeling socially disconnected, (2) feeling socially connected, and (3) feeling socially indifferent. Illustrative examples of each category are presented in Table 2 (and more details about the thematic content analysis have been previously presented in [3]). Participant responses were organized into these three categories by three trained researchers using Microsoft Excel (2022). Inter-rater reliability tests were conducted on 30% of the coded responses from the first two schools (i.e., 11.6% of the final sample). The kappas for the inter-rater reliability were calculated for each category (feeling socially disconnected, k = 0.97; feeling socially connected, k = 0.89; and feeling socially indifferent, k = 0.83), showing very high agreement among coders. Any coding disagreements were discussed until a consensus was reached. Results of the thematic coding of the qualitative data indicated that 24% of participants were categorized as feeling socially disconnected (*n =* 427), 64% as feeling socially connected (*n =* 1120), and 9% as feeling socially indifferent (*n =* 162) in the time of the COVID-19 global pandemic. There were 3% of participant responses (*n* = 44) that did not relate to their feelings of social connection in the time of the pandemic.

To further investigate predictors of feeling socially disconnected, we used a logistical regression analysis to test eight variables as predictors of the disconnected group. Results of the analysis are presented in Table 3, along with means, standard deviations, range, and alphas for all variables. Correlations among variables are presented in Table 4.

Findings from the binary logistic regression modeling for those who felt socially disconnected in the time of COVID-19 are shown in Table 5. Considering age, gender, school, and ethnicity (Model 1) (model fits: −2 log likelihood = 1850.25, Nagelkerke R^2^ = 0.011, χ^2^ (4) = 12.31, *p* = 0.015), only gender was significant. Entering depression, anxiety, and stress (Model 2) improved the model fit (model fits: −2 log likelihood = 1780.89, Nagelkerke R^2^ = 0.07, χ^2^ (7) = 81.66, *p* < 0.001), and depression was significant. Entering smartphone addiction (Model 3) again improved the model fit (model fits: −2 log likelihood = 1780.52, Nagelkerke R^2^ = 0.07, χ^2^ (8) = 82.04, *p* < 0.001), and depression (OR = 1.6) was significantly associated with social disconnection during the pandemic while smartphone addiction was not. The post hoc power analysis was conducted using G*Power version 3.1 [32] to determine the power; with a significance criterion of α = 0.05 and *N* = 1753, the power was 0.92 for the two-tailed, binary logistic regression.

## 4. Discussion

The COVID-19 global pandemic severely limited adolescents’ opportunities for face-to-face social contact, which meant that this demographic experienced an increase in mental health concerns, as well as increased reliance on technology, such as smartphones, to communicate with peers [1]. This increased reliance on technology led many to worry about adolescents’ smartphone addiction in the time of the pandemic. Given that mental health concerns and smartphone addiction are empirically established risk factors for adolescent loneliness and isolation, e.g., [10,22], the current study examined their relative risk in predicting adolescents’ feelings of social disconnection during the COVID-19 pandemic in Canada.

The key finding from this study was that smartphone addiction was not a significant predictor of feelings of social disconnection among Canadian adolescents during COVID-19. This finding should help appease some of the concern about adolescents’ increased use of technology during the pandemic. Indeed, this finding has important implications for how we understand the potential risks associated with excessive and compulsive smartphone use, suggesting that what we have deemed as a problematic pattern of usage (e.g., smartphone addiction) may in fact function as a protective factor against social disconnection when face-to-face social interactions are limited. It appears that these behaviors may play a somewhat adaptive role, in that they provide opportunities for adolescents to connect with others to meet their developmentally appropriate social needs. Thus, while previous work identified smartphone addiction as an important risk factor for loneliness and social isolation [10], this pattern was not present during COVID-19, when face-to-face social interactions were impeded. This finding has important implications for understanding patterns of technology engagement for youth who may be similarly constrained in meeting their social needs, for example, youth living in isolated rural communities, or for youth who are not able to have face-to-face interactions with their desired social groups (e.g., BIPOC youth living in primarily white neighborhoods; sexual minority youth whose in-person relationships do not support their orientation, etc.).

The findings from this work pointed to depression as a particularly salient risk factor for feelings of social disconnection during COVID-19. Indeed, depression was the only significant factor related to adolescents’ feelings of social disconnection in the current study, when controlling for adolescents’ stress, anxiety, smartphone addiction, and sociodemographic factors. This finding was contrary to our hypothesis that stress, anxiety, and depression would each be significantly associated with adolescents’ feelings of social disconnection in the time of the pandemic, given the tendency for youth with mental health concerns to withdraw socially from their peers [11]. Rather, the fact that only depression was a significant risk factor suggested that this type of mental health concern may make adolescents particularly vulnerable to feelings of social disconnection in times of limited face-to-face social interactions. This emphasizes the instrumental role of face-to-face interactions in fostering social connections for adolescents who struggle with depression and also highlights the importance of early identification and intervention for these youths.

### Limitations and Future Directions

Results from the current study added to the field of research on understanding the factors predicting feelings of social disconnection for Canadian adolescents during the COVID-19 pandemic. However, when interpreting these findings, there remained a few limitations to consider. First, all of our assessments were based on adolescent self-reports, which may have been biased in certain ways, e.g., recall bias and social desirability bias [33]. Therefore, future studies would benefit from utilizing interviews, parent reports, and teacher reports to assess adolescents’ mental health and smartphone addiction. Another limitation was the cross-sectional nature of the study, which means we did not have insight into the causal nature of the relationships between depression, smartphone addiction, and social disconnection. In addition, because the present study focused on adolescents, the findings may be limited in their applicability to young children or young adults. Finally, despite the significant findings, the explanatory power of this study remained low (Nagelkerke R^2^ = 0.07). We concentrated solely on the impact of mental health and smartphone addiction on adolescents’ feelings of socially disconnection; thus, future work should examine the role of other important factors such as personality traits [34] or emotion regulation [35] in predicting adolescents’ feelings of social disconnection.

## 5. Conclusions

The current study contributed to our understanding of the relative risk of adolescents’ mental health and smartphone addiction on their wellbeing in the time of the COVID-19 pandemic. Specifically, findings from this work indicated that depression was a significant risk factor for adolescents’ feelings of social disconnection during COVID-19, while smartphone addiction was not. This has important implications for the development of intervention and prevention efforts targeting adolescents’ wellbeing in times of limited face-to-face interactions (e.g., a global pandemic). Indeed, our findings suggested that these efforts should not be focusing on problematic patterns of smartphone use (i.e., smartphone addiction), but instead should aim to identify and support adolescents with depressive symptoms. In this way, our findings should help reduce some of the moral panic among parents, educators, and policymakers about the risks associated with a possible increase in smartphone addiction during the pandemic [4,5], recognizing that it is likely an adaptive response, and is not a significant predictor of adolescents’ social disconnection. Ideally, we can instead put our energies towards identifying those adolescents who are actually most at risk of experiencing feelings of social disconnection (i.e., those higher in depression).

## Figures and Tables

**Table 1:** Characteristics of participants.

Participants	Total (*n* = 1753)
Age in years (mean, standard deviation)	15.11 (1.46)
Grade (mean, standard deviation)	9.93 (1.35)
Gender ^a^	
Boy	862 (49.2%)
Girl	848 (48.4%)
Nonbinary	30 (1.7%)
Transgender	16 (0.9%)
Other/not sure	42 (2.4%)
Ethnicity ^b^	
East Asian	572 (32.6%)
Indigenous	43 (2.5%)
Latin American	60 (3.4%)
South Asian	395 (22.5%)
Southeast Asian	235 (13.4%)
West Asian	109 (6.2%)
White	404 (23.0%)
Other	27 (1.5%)

Note. ^a^ Some participants selected more than one gender. ^b^ Some participants selected more than one ethnic identification.

**Table 2:** Illustrative examples of themes identified through the thematic content analysis.

Theme	Example
Socially connected	“[…] The only thing COVID-19 changed was not being able to see my friends as much in real life and not being able to do sports. But through social media and playing video games I can still connect with my friends without actually seeing them”.
2. Socially disconnected	“Since the start of COVID-19, I have felt very lonely and disconnected from everyone else. I personally have felt like others have stopped talking to me or engaging with me outside of social media because they have better things to do or I am just not worth their time”.
3. Socially indifferent	“I feel it’s harder to talk to people just for safety’s sake but it hasn’t really changed much for me personally”.

**Table 3:** Descriptive statistics of predictors.

Variable	*n*	*M*	*SD*	*Min*	*Max*	Cronbach’s α
Depression	1751	3.85	0.91	1	5	0.91
Anxiety	1751	4.41	0.78	1	5	0.83
Stress	1751	3.08	1.29	1	5	0.86
Smartphone addiction	1703	2.38	0.79	1	6	0.81
Age	1743	15.11	1.46	11	20	*NA*
Gender	1753	0.57	0.71	0	3	*NA*
School	1753	3.16	1.40	1	5	*NA*
Ethnicity	1753	2.24	0.83	1	3	*NA*

Note. Ethnicity was coded as East Asian (1), White (2), and other (3) (including participants who selected more than one ethnicity: Black, Indigenous, Latin American, South Asian, Southeast Asian, and West Asian). Gender was coded as girls (1), boys (0), and others (2) (including participants who selected more than one gender: non-binary, transgender, and not sure). School was coded from 1 to 5; each number represents one school.

**Table 4:** Correlations among study variables.

	1	2	3	4	5	6
1. Depression	1					
2. Anxiety	0.49 **	1				
3. Stress	0.67 **	0.62 **	1			
4. Smartphone addiction	0.34 **	0.30 **	0.40 **	1		
5. Age	0.04	0.02	0.11 **	0.08 **	1	
6. School	−0.10 **	−0.10 **	−0.12 **	−0.06 *	−0.04	1

Note. * *p* < 0.05; ** *p* < 0.01; school was coded from 1 to 5; each number represents one school.

**Table 5:** Predictors of feeling socially disconnected.

	Model 1				Model 2				Model 3			
Variable	β	*OR*	*CI*	*p*	β	*OR*	*CI*	*p*	β	*OR*	*CI*	*p*
Gender	0.19 *	**1.2**	**[1.03–1.4]**	**0.01**	0.004	1.0	[0.85–1.2]	0.96	0.006	1.0	[0.85–1.2]	0.94
Age	0.03	1.0	[0.95–1.1]	0.46	0.013	1.0	[0.93–1.1]	0.76	0.014	1.0	[0.94–1.1]	0.73
School	−0.02	0.98	[0.89–1.0]	0.67	0.018	1.0	[0.93–1.1]	0.69	0.018	1.0	[0.93–1.1]	0.69
Ethnicity	−0.10	0.87	[0.75–0.99]	0.04	−0.18	0.84	[0.72–0.96]	0.23	−0.10	0.84	[0.72–0.96]	0.13
Depression					0.46 **	**10.6**	**[1.3–1.8]**	**<0.001**	0.46 **	**1.59**	**[1.3–1.9]**	**<0.001**
Anxiety					−0.03	0.97	[0.79–1.2]	0.79	−0.02	0.97	[0.79–1.2]	0.82
Stress					0.106	10.1	[0.97–1.3]	0.13	0.11	1.12	[0.97–1.3]	0.11
Smartphone addiction									−0.05	0.95	[0.81–1.1]	0.54

Note. *CI* is the 95% confidence interval for *OR*. * *p* < 0.01 ** *p* < 0.001. Ethnicity was coded as East Asian (1) (reference group), White (2), and others (3) (including participants who selected more than one ethnicity: Black, Indigenous, Latin American, South Asian, Southeast Asian, and West Asian). Gender was coded as girls (1) (reference group), boys (0), and others (2) (including participants who selected more than one gender: non-binary, transgender, and not sure). School was coded from 1 to 5; each number represents one school.

## Data Availability

Not applicable.
